# The Importance of Human–Computer Interaction in Radiology E-learning

**DOI:** 10.1007/s10278-015-9828-y

**Published:** 2015-10-13

**Authors:** Annemarie M. den Harder, Marissa Frijlingh, Cécile J. Ravesloot, Anne E. Oosterbaan, Anouk van der Gijp

**Affiliations:** Department of Radiology, Utrecht University Medical Center, P.O. Box 85500, E01.132, 3508 GA Utrecht, The Netherlands; Center for Research and Development of Education, University Medical Center Utrecht, Utrecht, The Netherlands

**Keywords:** Human–computer interaction, E-learning, Radiology, Education

## Abstract

With the development of cross-sectional imaging techniques and transformation to digital reading of radiological imaging, e-learning might be a promising tool in undergraduate radiology education. In this systematic review of the literature, we evaluate the emergence of image interaction possibilities in radiology e-learning programs and evidence for effects of radiology e-learning on learning outcomes and perspectives of medical students and teachers. A systematic search in PubMed, EMBASE, Cochrane, ERIC, and PsycInfo was performed. Articles were screened by two authors and included when they concerned the evaluation of radiological e-learning tools for undergraduate medical students. Nineteen articles were included. Seven studies evaluated e-learning programs with image interaction possibilities. Students perceived e-learning with image interaction possibilities to be a useful addition to learning with hard copy images and to be effective for learning 3D anatomy. Both e-learning programs with and without image interaction possibilities were found to improve radiological knowledge and skills. In general, students found e-learning programs easy to use, rated image quality high, and found the difficulty level of the courses appropriate. Furthermore, they felt that their knowledge and understanding of radiology improved by using e-learning. In conclusion, the addition of radiology e-learning in undergraduate medical education can improve radiological knowledge and image interpretation skills. Differences between the effect of e-learning with and without image interpretation possibilities on learning outcomes are unknown and should be subject to future research.

## Introduction

E-learning refers to the use of electronic media and network technologies for educational purposes and includes for example the use of audio, digital images, and web-based learning [1].

Due to the digital revolution, e-learning evolved rapidly in the past decades [2]. It is expected that e-learning will play an increasing role in future medical education and teaching strategies will change [3]. E-learning in general has several advantages over traditional, non-digital learning. First, e-learning gives learners the opportunity to learn at any time and at any location [4]. For example, e-lectures can be followed at home using the internet. Second, other types of study material, like animation and video clips, and interactive programs can be used. Third, the possibility to reach a high number of students might lead to cost reduction [3, 5].

Especially for radiology education, e-learning has a potential important benefit. E-learning makes human–computer interaction possible. The two most important forms of human–computer interaction in radiology are navigation (scrolling through stack of images in different planes) and manipulation (adjusting contrast setting, rotating 3D models) [6]. Computed tomography (CT) and magnetic resonance imaging (MRI) are widely used in almost all medical specialties and viewed as a stack of images (volumetric image) instead of single images printed next to each other. Digitalization and the emergence of PACS (Picture Archiving and Communication Systems) made radiological images, including all image manipulation tools, available for all in-hospital doctors. Therefore, it is important that all medical students learn how to interpret radiological images and understand the relation between anatomical and pathological structures. Cognitive processes in volumetric and 2D image interpretation differ substantially, which makes it important for students to learn to interpret both image types [7]. A first step can be the use of videos of volumetric image stacks. However, ultimately human–computer interaction with the images should be possible since this is more representative to clinical practice and more reliable than tests with 2D CT images [7].

E-learning has the potential to improve radiology education because it allows for authentic image manipulation, for example, scrolling through CT and MRI scans, which helps students to understand the 3D relations between anatomical and pathological structures [8]. In this study, we evaluate the emergence of image interaction in radiology e-learning programs in radiology education literature and the existing evidence of effects of radiology e-learning programs with and without image interaction possibilities on learning outcomes and perspectives of medical students and teachers.

## Methods

### Search

The electronic databases PubMed, EMBASE, Cochrane, ERIC, and PsycInfo were searched. All medical, psychological, and educational electronic databases were used to perform an extensive search because the topic of this review can be categorized in different databases. The terms education, radiology, e-learning, and synonyms of these words were combined. The search syntax is provided in Table 1. Duplicates were removed, and the articles were screened by two authors (AH and MF) on title and abstract using predefined in- and exclusion criteria. Relevant articles were screened on full text and included if eligible. Articles concerning evaluation of radiologic e-learning programs for undergraduate medical students were included. Exclusion criteria were (1) language other than English, (2) posters or congress reports, (3) no full text availability, (4) concerning nuclear medicine, (5) only description of the e-learning program and no evaluation, and (6) articles that included other study populations (e.g., residents, radiologists, non-medical students) and did not report outcomes separately for undergraduate medical students. A manual search was performed by authors with expertise on e-learning in radiology. Finally, reference lists of reviews retrieved through the search were screened for additional articles.

**Table 1:** Search syntax

Databases searched	Search terms entered into databases	Relevant MeSH terms/Emtrees/subject headings used
PubMed In title and abstract EMBASE In title and abstract Cochrane In title and abstract ERIC In title and abstract PsychInfo In title and abstract	Radiol* OR radiology AND Elearning OR e-learning OR e learning OR technol* enhanced learning OR technol* enhanced teaching OR webbased learning OR webbased teaching OR web-based learning OR web-based teaching OR web based learning OR web based teaching OR electronic learning OR electronic teaching OR online learning OR online teaching AND Relevant MeSH terms/emtrees/subject headings	PubMed MeSH terms Education, Distance; Radiology EMBASE Emtree terms Radiology PsychInfo Subject headings Distance Education; Electronic learning; Radiology ERIC Subject headings Distance Education; Electronic learning; Radiology

### Data Extraction and Quality Assessment

Two authors (AH and MF) independently extracted data and assessed quality of the included articles. In case of discrepancy, consensus was reached between authors. Study quality was assessed using the Medical Education Research Study Quality Instrument (MERSQI), developed to measure the quality of educational research studies [9]. The MERSQI also includes evaluation of the Kirkpatrick level which consists of four levels of evaluation, namely (1) reaction, (2) learning, (3) behavior, and (4) results [10, 11]. Outcomes were (1) the effect of e-learning on learning outcomes, (2) students’, and (3) teachers’ perspectives on e-learning.

## Results

**Fig. 1:**
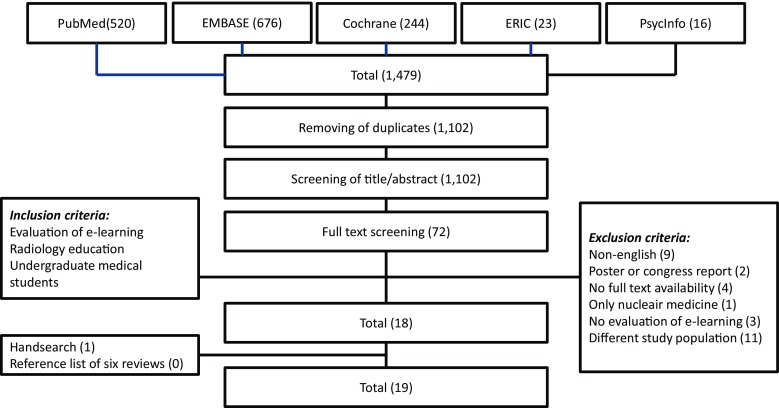
Flowchart of study inclusion

The search yielded 1479 articles of which 1102 articles remained after removing duplicates. A flowchart is provided in Fig. 1. Nineteen relevant articles were included for further analysis. Baseline characteristics of the included studies are shown in Table 2. Included studies were published between 2000 and 2013. Most studies were performed in the USA, Germany, or UK and included 26 to 687 students. Five studies used a control group of students. Imaging modalities used were CT (*n* = 14), MRI (*n* = 9), X-ray (*n* = 11), angiography (*n* = 5), and ultrasound (*n* = 6). Twelve studies (63 %) used a web-based e-learning program. The evaluated e-learning course was mandatory in 26 % (five studies).

**Table 2:** Characteristics of the included studies

	Year of publication	Country	Undergraduate year	Number of participants of e-learning evaluation (total participants)	Number of students in control group (*n*)	Imaging modalities					Mandatory e-learning course (yes/no/?)	Online(yes/no/?)	Image viewing mode
						CT	MRI	X-ray	Angiography	Ultrasound			
Arya et al. [14]	2013	US	3	47 (?)	NA	+	+	−	+	+	No	Yes	S
Bohl et al. [22]	2011	US	1–4	78 (193)	NA	+	−	−	−	−	No	Yes	T
Chorney et al. [29]	2011	US	3–4	77 (687)	NA	+	+	+	+	+	Yes	Yes	T
Davison et al. [25]	2000	US	?	76 (76)	NA	?	?	?	?	?	No	Yes	T
Dugas et al. [30]	2001	Germany	3	101 (101)	NA	+	+	+	−	−	Yes	Yes	T
Ernst et al. [15]	2003	US	1	? (?)	NA	+	?	+	?	?	No	No	S
Gotthardt et al. [28]	2006	Germany	3	257 (276)	NA	+	?	?	?	?	?	Yes	T
Howlett et al. [16]	2011	UK	5	? (211)	NA	+	?	+	?	?	Yes	Yes	S
Jafri et al. [17]	2008	US	2	89 (166)	NA	+	+	+	+	+	?	No	S
Leong et al. [27]	2012	Ireland	4	126 (127)	NA	−	−	−	−	−	No	Yes	NA
Mahnken et al. [12]	2011	Germany	4	64 (64)	32	+	+	+	−	+	Partly	Yes	T
Maleck et al. [13]	2001	Germany	3	85 (192)	107	−	−	+	−	−	Yes	?	T
Petersson et al. [18]	2009	Sweden	?	33 (?)	42	+	+	−	−	−	No	Yes	T
Rengier et al. [19]	2013	Germany	4-5	25 (27)	NA	+	+	−	−	−	No	No	S
Shaffer et al. [20]	2004	US	1	266 (340)	141 (168)	+	+	?	?	+	No	Yes	S
Tachakra et al. [26]	2000	UK	?	26 (26)	NA	−	−	+	−	−	?	No	T
Turmezei et al. [21]	2009	UK	1	141 (260)	NA	+	+	+	+	+	No	No	S
Wagner et al. [24]	2005	Worldwide	?	515 (515)	NA	+	?	+	+	?	No	Yes	T
Webb et al. [23]	2013	Australia	1	116 (167)	NA	−	−	+	−	−	No	No	T

*US* United States, *?* not provided, *NA* not applicable, *+* used in the course, *−* not used in the course, *T* tile, *S* stack

### Quality Assessment

The MERSQI score of the included articles is provided in Table 3. The Kirkpatrick level, which is included in the MERSQI score assessment, was 1 in 11 of the studies. This means that only the perception of students of the e-learning program was investigated. Seven studies also evaluated the effect of the e-learning program on knowledge and skills (Kirkpatrick level 2). The MERSQI score varied from 7 to 14.5 (out of 18). Most studies did not randomize groups (except for the studies of Mahnken et al. [12, 13] and Maleck et al. [13]), and none of the studies assessed the effect of e-learning on student behavior or patient outcomes.

**Table 3:** MERSQI score assessment. The maximum achievable score for each row is 18

	Study design				Sampling							Type of data		Validity of evaluation instrument									Data analysis				Outcomes				Total score
	Single group cross-sectional or single group posttest only	Single group pretest and posttest	Nonrandomized, 2 group	Randomized controlled trial	No. of institutions studied			Response rate, %				Assessment by study participant	Objective measurement	Internal structure			Content			Relationships to other variables			Appropriateness of analysis		Complexity of analysis		Satisfaction, attitudes, perceptions, opinions, general facts	Knowledge, skills	Behaviors	Patients/health care outcome	
					1	2	>2	Not applicable	<50 or not reported	50–74	≥75			Not applicable	Not reported	Reported	Not applicable	Not reported	Reported	Not applicable	Not reported	Reported	Data analysis inappropriate for study design or type of data	Data analysis appropriate for study design and type of data	Descriptive analysis only	Beyond descriptive analysis					
Score	1	1.5	2	3	0.5	1	1.5		0.5	1	1.5	1	3	-	0	1	-	0	1	-	0	1	0	1	1	2	1	1.5	2	3	
Arya et al. [14]	1				0.5				0.5			1			0			0			0			1		2	1				7
Bohl et al. [22]	1				0.5				0.5			1			0				1			1		1	1		1				8
Chorney et al. [29]	1				0.5				0.5			1			0				1			1		1	1		1				8
Davison et al. [25]			2		0.5						1.5		3		0			0			0			1		2		1.5			11.5
Dugas et al. [30]	1				0.5						1.5	1			0				1		0			1	1		1				8
Ernst et al. [15]	1				0.5				0.5			1			0				1		0			1	1		1				7
Gotthardt et al. [28]	1				0.5						1.5		3		0				1			1		1	1			1.5			11.5
Howlett et al. [16]	1				0.5				0.5			1			0				1		0			1	1		1				7
Jafri et al. [17]	1				0.5					1		1			0				1		0			1	1		1				7.5
Leong et al. [27]		1.5			0.5						1.5		3		0				1		0			1		2		1.5			12
Mahnken et al. [12]				3	0.5						1.5		3		0				1			1		1		2		1.5			14.5
Maleck et al. [13]				3	0.5						1.5		3		0				1		0			1		2		1.5			13.5
Petersson et al. [18]			2		0.5				0.5				3		0				1		0			1	1			1.5			10.5
Rengier et al. [19]		1.5			0.5						1.5		3		0				1		0			1		2		1.5			12
Shaffer et al. [20]	1				0.5						1.5	1			0				1		0			1	1		1				8
Tachakra et al. [26]	1				0.5						1.5	1			0				1		0			1		2	1				9
Turmezei et al. [21]	1					1				1		1			0				1		0			1	1		1				8
Wagner et al. [24]	1						1.5				1.5	1			0				1			1		1	1		1				10
Webb et al. [23]		1.5			0.5					1		1			0				1			1		1	1			1.5			9.5

### Image Interaction Possibilities

In seven studies, the e-learning program offered students the possibility of viewing a stack of images [14–21]. Of which two studies used videos, i.e., scroll rated was fixed [15, 16], while in the other five studies, students could scroll through the images at their own pace [14, 17–21]. In one study, students were allowed to rotate 3D models of vessels derived from volumetric images [18]. Two studies [17, 19] additionally offered the possibility to adjust contrast settings.

### Effect of Radiology E-learning on Learning Outcomes

The reported outcomes for each study are provided in Table 4. Eight studies (42 %) investigated the effect of radiology e-learning on learning outcomes, of which two studies concerned e-learning with image interaction possibilities. Learning effects were measured with pre- and post-course radiology skills tests, and all studies reported improved test results after the e-learning course. Three studies compared the test results with a control group, including one study using e-learning with image interaction possibilities [18], and found a higher improvement in knowledge in students exposed to e-learning compared to a control group [12, 13, 18].

**Table 4:** Reported outcomes for each study

	Year of publication	Effect on learning outcome / test results	Students perspectives													Teacher perspectives			
			Image quality	Clear instructions/user friendly	Appropriate level of difficulty	Good alternative to didactic lectures	Clinically relevant	Improved recognition of pathology	Improved understanding of anatomy/radiology	Too much/too detailed information	Not relevant	Increased interest in radiology	Learning from home/other places	Learner isolation	Covers larger areas of knowledge	Less administrative tasks	Decrease in costs	Covers larger areas of knowledge	Technical problems
Arya et al. [14]	2013		+	+			+		+	+									
Bohl et al. [22]	2011			+		+	+	+	+		+								
Chorney et al. [29]	2011				+				+										
Davison et al. [25]	2003	+				+										+			
Dugas et al. [30]	2001		+	+					+										
Ernst et al. [15]	2003			+													+		
Gotthardt et al. [28]	2006	+							+				+						+
Howlett et al. [16]	2011		+		+	+	+	+					+		+			+	
Jafri et al. [17]	2008			+	+		+			+									
Leong et al. [27]	2012	+			+	+			+			+	+	+					+
Mahnken et al. [12]	2011	+																	
Maleck et al. [13]	2001	+				+			+										
Petersson et al. [18]	2009	+				+													
Rengier et al. [19]	2013	+							+										
Shaffer et al. [20]	2004																		+
Tachakra et al. [26]	2000		+			+			+										
Turmezei et al. [21]	2009			+			+		+										+
Wagner et al. [24]	2005		+	+			+	+											
Webb et al. [23]	2013	+	+	+	+		+	+	+			+	+						+
Total		8	6	8	5	7	7	4	11	2	1	2	4	1	1	1	1	1	5

The study of Petersson et al. [18] investigated the value of an additional 3D radiological anatomy e-learning tool for students taking vascular anatomy courses. Student test scores on 3D anatomical knowledge of peripheral vessels improved significantly after introduction of the additional e-learning tool compared to test scores of control groups from a year before who only took the traditional vascular anatomy courses. Radiological knowledge of neurovascular anatomy did not significantly improve after introducing the e-learning course.

Maleck et al. [13] randomized students in four groups: group 1 and 2 were presented with e-learning cases, respectively, with and without interactive elements; group 3 with paper-based cases with interactive elements, and a control group was not exposed to any cases. Interactive elements comprised questions guiding students through the cases and did not relate to image interaction in this case. All students had the opportunity to attend radiology lectures on a voluntary basis. Pre- and post-course tests with questions related to radiological knowledge and image interpretation skills were used. Both multiple choice questions and free-text questions were used related to radiographs projected with a slide projector. The authors found significant improvement in knowledge and X-ray image interpretation skills in both e-learning groups and in the paper-based group in contrast to the control group who showed no significant improvement. Most improvement in image interpretation skills was found in e-learning group 1, and most improvement in knowledge was found in e-learning group 2, though it was not reported if these differences were significant.

Mahnken et al. [12] provided e-learning with radiological cases and expert feedback and compared this to a group without access to the e-learning environment. Image interaction was not possible. All students followed an internship in radiology, so the control group was exposed to radiology as well but without access to the additional e-learning program. Learning effects were measured with a pre- and post-course radiology knowledge test based on the learning objectives of the internship and the e-learning content. Knowledge improvement in the e-learning group was higher, but this difference was not significant. None of the studies compared the learning effect of e-learning alone in comparison to traditional learning.

### Students’ Perspectives on E-learning with Image Interaction Possibilities

Most studies reported that the possibility of image interaction was advantageous to students. For example, Ernst et al. [15] introduced a CD-ROM with stack viewing in their learning program, which was found to be a useful addition to hard copy images by 96 % of students. In the study of Arya et al. [14], an e-learning module with stack image viewing was compared to three other methods: a 3D anatomic model, a poster with tiled images, and an ultrasound station. The e-learning module was found to be most helpful in recall of anatomic principles and was perceived to be, together with the 3D anatomic model, more effective in improving the comprehension of 3D physical relationships than the other two methods. The e-learning station and the ultrasound station were thought to represent more clinically relevant material than the other two. Overall, the 3D model was found to be most valuable by students. According to a qualitative evaluation of an e-learning program using 2D images and videos of stacks, students particularly appreciate stacks of cross-sectional images for radiology learning [16]. In another study with scrollable images, 3 % of the participants suggested to add more image interaction possibilities, such as changing viewing direction [21]. One negative comment concerning image interaction was reported and concerned the lack of facilitators to assist in navigating through the scrollable images [17].

In the study of Bohl et al. [22], students did not have the opportunity to use image interaction. However, some students mentioned that it would be better to use actual software for practice which allows image interaction.

### Students’ Perspectives on Radiology E-learning in General

Nearly all studies (89 %) investigated the perspectives of students on radiology e-learning in general. The quality of the images used in e-learning was rated high in six studies. Only one study with X-ray images reported that a few students (4 %) found that the image quality should be improved [23].

Seven studies described that students found that they could use the knowledge gained from the e-learning programs in clinical practice [14, 16, 17, 21–24]. On the other hand, students also experienced that the information in the e-learning course was sometimes too much, too detailed, or not relevant for the post-course assessment [14, 17, 22].

Seven studies addressed the question if e-learning could be an alternative to traditional lectures. In three studies, the e-learning program was preferred over traditional learning [13, 22, 25]. Two studies reported that students found e-learning alone not suitable but preferred e-learning as an addition to traditional lectures [16, 26]. Traditional lectures were preferred over e-learning by the majority of students in two studies [18, 27]. One of these studies mentioned that students missed the student–teacher interaction [27]. However, this study was essentially different from the other included studies as it investigated the use of e-learning in radiation protection education, and therefore, radiological images did not play a central role. The e-learning course existed of information hyperlinked to webpages and images from the Internet.

In 11 studies, students mentioned that e-learning improved their understanding of radiology and anatomy [13, 14, 19, 21–23, 26–30]. Specifically, they experienced improved recognition of pathology on radiological images [22–24]. Students also mentioned that they were more interested in radiology after the e-learning course [23, 27]. Students liked the possibility to study from home and found that e-learning made it possible to cover larger areas of knowledge in a short time frame [16, 23, 27, 28].

### Teacher Perspectives

Advantages of e-learning that were experienced by authors were less administrative tasks and a decrease in costs [15, 16, 25]. Administrative tasks reduced because exams could be reviewed electronically [25]. Ernst et al. [15] experienced a decrease in costs because CD-ROMs containing both the syllabus and radiology images were used instead of a printed syllabus. In several studies, technical problems were experienced, making it temporarily impossible to access the e-learning program [20, 21, 23, 27, 28].

## Discussion

E-learning offers the opportunity to let users interact with radiological images but less than half of the included studies took advantage of this possibility. Most studies only used static images which also could be used in traditional learning. The image interaction possibilities varied from videos of an image stack to scrolling freely through stacks of images in multiple directions and contrast settings. Students found image interaction possibilities valuable and encourage its use in e-learning programs. The effect of e-learning with and without image interpretation possibilities on learning outcomes was not compared.

Radiology e-learning can improve learning outcomes of students. Some results suggest that the addition of e-learning to traditional learning can lead to improved radiological knowledge and image interpretation skills. Students’ perspectives of e-learning are generally positive, and students feel that their knowledge and understanding of radiology improves by using e-learning. However, we did not find evidence to conclude that e-learning methods are superior to traditional teaching methods or that e-learning methods could replace traditional lectures because none of the studies compared both methods individually. Students’ opinions about this topic were diverse. In three studies, students preferred e-learning over traditional lectures, while in two studies, traditional lectures were preferred. Two other studies reported that students preferred e-learning as an addition to traditional lectures.

The level of evidence of the included studies was relatively low. First, a lot of studies investigate students’ perspectives on e-learning, and there was relatively few evidence of the effect on learning outcomes. This is reflected in the low Kirkpatrick levels of the investigated studies, namely only level 1 and level 2. It would be interesting to know if e-learning also leads to improved image interpretation in clinical practice (Kirkpatrick level 4). However, this would be challenging to investigate because improved performance in clinical practice usually cannot be traced to a single educational component. An improvement in performance is the result of different educational activities and is also influenced by what students learn on their own initiative by reading books, for example. Most studies did not compare their results to a control group, and in only one study, students were randomized in groups. Moreover, most studies were descriptive and did not go beyond descriptive analysis. As most comparative studies added e-learning to traditional learning, part of the effect of e-learning can possibly be explained by the benefit of additional education and extra study time. Another limitation was the large diversity between the included studies. For example, each study used a different e-learning and software program. Further, some studies used small study populations.

The current study included a thorough and systematic search; however, there are a few limitations. The manual search identified an additional article which was not found in the original search. The reason was that this article did not name their new software for radiology education as e-learning, and therefore, this article did not show up in our initial search. There might probably be more studies evaluating the effect of an e-learning tool that were not detected by our search. Further, local unpublished initiatives of certain e-learning programs were not included in the search, but they might contain very valuable information. To further evaluate the effect of e-learning, it might be useful to also investigate the evidence for e-learning in related fields. In this study, only radiology was investigated because radiology is different due to cross-sectional imaging techniques and the transformation to digital reading of radiological images.

According to this review study, no studies investigated the difference in learning effect between radiology e-learning with and without image interaction possibilities. In the assessment literature, however, tests with and without image interaction possibilities were compared, and tests using stack images were found to be more reliable and more representative for clinical practice compared to tests with 2D images [7]. Since all in-hospital doctors have the ability to scroll through stacks of images and apply other image manipulation possibilities in daily practice, and this requires different cognitive processes than viewing 2D images [31], medical students could benefit from exposure to this aspect of image interpretation. Further, radiological anatomy test scores with stack viewing and image manipulation possibilities correlated significantly to scores on human cadaver anatomy tests, while scores on tests without stack viewing did not correlate to human cadaver test scores [7]. This might indicate that in a radiology-anatomy course, it would be preferable to use radiological image with stack viewing and other image manipulation tools.

Evidence for effects of e-learning in medical education was previously reported in another review study, concerning e-learning methods in continuing medical education for health care professionals [32]. In this context, in contrast to the undergraduate radiology domain, several studies compared web-based programs to traditional lectures in randomized controlled trials. Traditional teaching was found to be as effective as internet-based learning programs [32]. A systematic review investigating online learning for undergraduate nurse education also found that online learning was as effective as traditional learning [33]. Some studies reported benefits of web-based programs in comparison to print materials with respect to learning outcomes, learning efficiency, and learners’ perspectives. However, we cannot directly transfer these results to undergraduate radiology education, as the content of the learning material, learning goals, learner characteristics, and education levels substantially differ. Randomized controlled trials are therefore needed to compare the effect of e-learning and traditional learning in undergraduate radiology education. In addition, we found little evidence for behavioral change in clinical practice as a result of e-learning [32].

## Summary

E-learning can improve radiological knowledge and image interpretation of undergraduate medical students, in addition to traditional learning. Image interaction possibilities largely varied among studies and were encouraged by students. The use of image interaction in radiology e-learning might be beneficial for medical students.
